# A Lightweight Automatic Wildlife Recognition Model Design Method Mitigating Shortcut Learning

**DOI:** 10.3390/ani13050838

**Published:** 2023-02-25

**Authors:** Yujie Zhong, Xiao Li, Jiangjian Xie, Junguo Zhang

**Affiliations:** 1 School of Technology, Beijing Forestry University, Beijing 100083, China; 2 Research Center for Biodiversity Intelligent Monitoring, Beijing Forestry University, Beijing 100083, China

**Keywords:** automatic wildlife recognition, shortcut learning, data augmentation, model compression

## Abstract

**Simple Summary:**

Due to the complexity of the wild environment, wildlife recognition based on camera trap images is challenging. Indeed, as the backgrounds of images captured from the same infrared camera trap are rather similar, shortcut learning of recognition models are produced, resulting in reduced generality and poor recognition model performance. Therefore, we propose a data augmentation strategy that integrates image synthesis (IS) and regional background suppression (RBS). This strategy alleviates a model’s focus on the background, guiding it to focus on the wildlife in order to improve the model’s generality, resulting in better recognition performance. Furthermore, in order to offer the lightweight recognition model for deep learning-based real-time wildlife monitoring on edge devices, we developed a model compression strategy that combines adaptive pruning and knowledge distillation. The produced lightweight model can reduce the computational effort of wildlife recognition with less loss of accuracy and is beneficial for real-time wildlife monitoring with the use of edge intelligence.

**Abstract:**

Recognizing wildlife based on camera trap images is challenging due to the complexity of the wild environment. Deep learning is an optional approach to solve this problem. However, the backgrounds of images captured from the same infrared camera trap are rather similar, and shortcut learning of recognition models occurs, resulting in reduced generality and poor recognition model performance. Therefore, this paper proposes a data augmentation strategy that integrates image synthesis (IS) and regional background suppression (RBS) to enrich the background scene and suppress the existing background information. This strategy alleviates the model’s focus on the background, guiding it to focus on the wildlife in order to improve the model’s generality, resulting in better recognition performance. Furthermore, to offer a lightweight recognition model for deep learning-based real-time wildlife monitoring on edge devices, we develop a model compression strategy that combines adaptive pruning and knowledge distillation. Specifically, a student model is built using a genetic algorithm-based pruning technique and adaptive batch normalization (GA-ABN). A mean square error (MSE) loss-based knowledge distillation method is then used to fine-tune the student model so as to generate a lightweight recognition model. The produced lightweight model can reduce the computational effort of wildlife recognition with only a 4.73% loss in accuracy. Extensive experiments have demonstrated the advantages of our method, which is beneficial for real-time wildlife monitoring with edge intelligence.

## 1. Introduction

Accurate wildlife density and abundance monitoring assists in analysis of the causes of biodiversity loss and assessment of the impacts of conservation measures [1]. According to the International Union for Conservation of Nature (IUCN), up to 17,000 species are considered as “data deficient” [2]. Therefore, there is an urgent need for effective large-scale wildlife monitoring systems with great spatiotemporal resolution. Camera traps have become an essential tool for wildlife monitoring in the recent decades, collecting huge amounts of data every day [3]. Because manual annotation of such huge amounts of data is time-consuming, automatic wildlife recognition is an appealing method for analyzing these data [4,5]. Deep learning methods have recently emerged as the dominant method for automatically recognizing wildlife. Xie et al. [6] introduced the SE-ResNeXt model for recognizing 26 wildlife species in the Snapshot Serengeti dataset, with the highest Top-1 and Top-5 accuracy levels of 95.3% and 98.8%, respectively. Silva et al. [7] utilized ResNet50 to classify different species of “bush pigs”, with a best accuracy of 98.33%. Nguyen et al. [8] designed Lite AlexNet to identify the three most common species in South Central Victoria, Australia, with an accuracy of 90.4%. Tan et al. [5] compared three mainstream detection models, YOLOv5, FCOS, and Cascade R-CNN, on the Northeast Tiger and Leopard National Park wildlife image dataset (NTLNP dataset). YOLOv5, FCOS, and Cascade R-CNN all obtained high average precision values: >97.9% at mAP_0.5 and >81.2% at mAP_0.5: 0.95. These studies indicate that deep convolutional neural networks (CNNs) can perform well in wildlife recognition.

Although existing automatic wildlife recognition methods have achieved higher and higher accuracy, these models’ capacity to generalize across diverse datasets is not as strong as it could be. Geirhos et al. [9] suggested that the aforementioned issue might be attributed to shortcut learning, in which these models tend to learn simple decision rules during training. These learned decision rules can only perform well on datasets that are independent and identically distributed (i.i.d.). In a non-independent and identically distributed (non-IID) dataset, however, performance deteriorated. Because camera traps are typically deployed in fixed locations, wildlife monitoring images appear to have similar backgrounds over time. This demonstrates a strong coupling link between wildlife and their backgrounds, providing shortcuts for a deep learning model to recognize wildlife via the backgrounds. These models may fail to recognize the same species with different backgrounds given the shortcut-learned decision rules. To increase the accuracy and generalization capabilities of wildlife recognition models, shortcut learning must be avoided.

Many strategies for mitigating shortcut learning have been investigated. Szegedy et al. [10] proved that data distribution has a direct impact on deep neural network learning and generated adversarial samples by adding perturbations to the data to avoid shortcut learning. Cubuk et al. [11] improved the generalization of object recognition models by augmenting data with information, geometric distortion, and color distortion. Arjovsky et al. [12] used causality to distinguish the false correlation and the interest region in the sample and then proposed invariant risk minimization (IRM), a novel learning framework that can estimate nonlinear, invariant, causal predictors across different training environments, allowing for out-of-distribution generalization. Finn et al. [13] utilized meta-learning to train a model on multiple learning tasks, resulting in high generalization performance with only a modest number of new training samples. Overall, data augmentation is a a simple and effective technique for avoiding shortcut learning.

Furthermore, it is beneficial to conduct recognition directly on a camera trap to improve the effectiveness of wildlife monitoring [14]. Due to the limited computing capability and memory of camera traps, a lightweight recognition model is required. Model compression is a commonly used method to generate a lightweight model [15]. Knowledge distillation [16] transfers the knowledge gained by a large teacher network to a small student network without losing validity, allowing model compression to be realized. The structure of the student model is crucial to the success of compression and the performance of the compressed model. Wen et al. [17] suggested a structural sparse learning approach for obtaining the student network from a large CNN, which accelerated AlexNet by 5.1 and 3.1 times on CPU and GPU, respectively, with only a 1% drop in accuracy. Rather than compressing the teacher network, Du et al. [18] selected a shallow reference model as the student network, which was then combined with a random forest model to generate more precise probability values for each class. Crowley et al. [19] separated the normal convolution of the teacher model into different groups of point-wise convolutions to construct several student models with test errors ranging from 5.0% to 7.87%.

Model pruning is another popular model compression method that can significantly reduce the number of parameters by removing certain parts of the model [20]. It is regarded as an effective method for achieving a student network. There are two types of pruning, i.e., unstructured pruning and structured pruning. Unstructured pruning approaches remove weights on a case-by-case basis, e.g., HashedNet [20], which uses a hash function to randomly group network weights, then allows weights in the same group to share parameters to minimize model complexity. Structured pruning methods remove weights in groups, such as a channel or a layer, and are often more efficient than unstructured pruning methods. Li et al. [21] removed convolution kernels with low effect on network accuracy and then retrained the pruned model to boost accuracy. Luo et al. [22] developed a pruning technique for ThiNet based on a greedy strategy. By considering network pruning to be an optimization issue, the statistical information obtained from the next layer’s input–output relationship is utilized to determine how to prune the present layer. ThiNet can reduce the parameters and FLOPs of ResNet-50 by more than half, whereas top-5 can only reduce them by 1%. He et al. [23] utilized a channel pruning approach to reduce the channel information in the input feature map before retaining it in the output feature map by adjusting the weights. Under a scenario with five-fold acceleration, the approach only degrades the accuracy of a VGG16 network by 0.3%. To achieve a high compression ratio, Jin et al. [24] proposed a hybrid pruning strategy that integrated kernel pruning and weight pruning. Aghli et al. [25] first employed the weight pruning method to create a student model, then knowledge distillation was achieved by minimizing the cosine similarity between the layers of the teacher and student networks. When compared to the state-of-the-art methods, higher compression rates can be achieved with comparable accuracy. All in all, it appears that the combination method of model pruning and knowledge distillation is more suited for generating a lightweight student network with acceptable accuracy.

**Figure 1:**
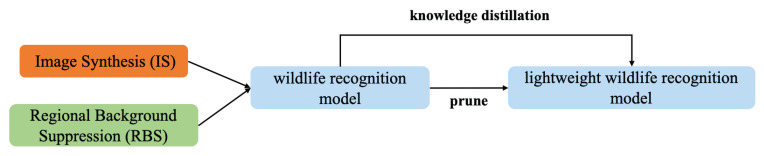
The technological framework for the design of the lightweight automatic wildlife recognition model.

In this paper, we propose a lightweight automatic wildlife recognition model design method that avoids shortcut learning. To the best of our knowledge, this is the first work that focuses on the shortcut learning of camera trap image recognition. First, two data augmentation strategies—image synthesis (IS) and regional background suppression (RBS)—are introduced in order to prevent the wildlife recognition model from shortcut learning and improve its performance. The Resnet50-based wildlife recognition model is then pruned with the genetic algorithm and adaptive BN (GA-ABN) to construct the student model. Finally, utilizing the Resnet50-based wildlife recognition model as a teacher model, knowledge distillation is employed to fine-tune the student model, yielding a lightweight automatic wildlife recognition model. The technological framework for the design of the lightweight automatic wildlife recognition model is depicted in Figure 1.

To summarize, the contribution of this work is two-fold:

(1) We introduce a novel mixed data augmentation method that combines IS and RBS to mitigate shortcut learning in wildlife recognition.
(2) We propose an effective model compression strategy based on GA-ABN for adaptively reducing the redundant parameters of a large wildlife recognition model while maintaining accuracy.

## 2. Materials and Methods

### 2.1. Wildlife-6 Dataset

From 2010 to 2019, we utilized infrared camera traps to collect wildlife monitoring images in Saihanwula National Nature Reserve in Inner Mongolia [26]. We selected images of six common species: red deer, goral, roe deer, lynx, badger, and wild boar [27]. After discarding images that were falsely triggered or damaged, the LabelMe software [28] was used to annotate the remaining images with bounding boxes and categories. The annotated dataset is denoted as Wildlife-6. Details of Wildlife-6 are shown in Table 1.

### 2.2. Mixed Data Augmentation

In this section, a mixed data augmentation method combining IS and RBS is proposed to diversify the background.

#### 2.2.1. Image Synthesis Based on Random Pasting

**Figure 2:**
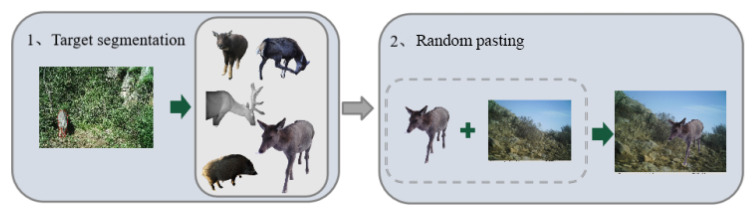
Image synthesis method.

We present an image synthesis method for generating new training samples by randomly pasting wildlife instances into background images of camera traps. Figure 2 shows the image synthesis procedure. There are two main stages: (1) target segmentation and (2) random pasting.

In the target segmentation stage, wildlife instances are extracted using the weakly supervised semantic segmentation model Inter-pixel Relation Network (IRNet) [29]. First, we trained the ResNet50-based wildlife recognition model and generated the class activation mapping (CAM). The confidence region of CAM was then obtained using the DenseCFR method, which was based on the thresholding of the foreground and background. The random walk method was applied to select pairs of points in the confidence region, which were then input into the IRNet for training. During training, IRNet learns to produce class boundary maps to determine class boundaries and compute class centroids. The optimal class centroids are achieved by iteration. Finally, the random walk algorithm is used to compute the attention scores of the pixel points in the CAM, as well as the domain propagation of the instance image based on the semantic affinity between adjacent pixels, to obtain the entire instance region, i.e., the wildlife instance.

**Figure 3:**
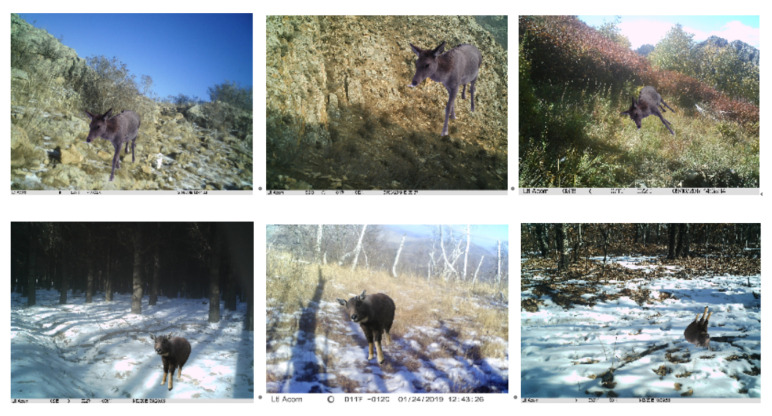
Synthetic samples.

Furthermore, in the random pasting stage, the extracted wildlife instances are randomly rotated, resized, and then pasted to a random background image to disrupt their inherent directional properties and reduce the object’s dependency on the entrenched scene. Figure 3 depicts synthetic sample instances.

#### 2.2.2. Regional Background Suppression

We propose a target-guided background suppression method to guide the network to focus on the foreground (wildlife). Unlike the cutout method, our method only randomly suppresses the background regions. The background suppression is divided into the following stages.

To begin, separate the image into foreground (wildlife) and background. To properly extract the foreground, we label the wildlife in the image with a bounding box, and the region outside the bounding box is regarded as background.

Then, given a random starting point and height–width ratio, we generate a rectangular mask $m_{b}$ with all values equal to 0. With the bounding box region $m_{f}$, it is critical to guarantee that $m_{f}\cap m_{b}=\emptyset$. In this case, the random mask suppresses background features, forcing the network to focus on foreground (wildlife) features. The generated images with background occlusion may be utilized not only to model occlusion phenomena in the wild, but also to help the model focus on more foreground (wildlife) information.

### 2.3. Lightweight Method for Automatic Wildlife Recognition Model

#### 2.3.1. Pruning Method Based on Genetic Algorithm and Adaptive Batch Normalization

A structural prune method based on GA-ABN is presented to construct the student model. First, the genetic algorithm(GA) is applied to obtain an optimal pruning strategy. To begin, a set of sub-networks are created as the initial population using a random sampling method. A fitness function is introduced to evaluate each sub-network in the initial population and is shown in Equation (1).

(1) $$f_{j}=\frac{n_{j}}{p_{j}/p_{0}}$$

where $n_{j}$ is the validation accuracy of the *j*-th sub-network, and the parameters $p_{j}$ and $p_{0}$ represent the number of the *j*-th sub-network and the initial network, respectively.

The fitness score of each sub-network is then determined in the selection stage. The top 20 sub-networks with the highest fitness scores are chosen. During the crossover mutation step, a new population with 100 new sub-networks is generated based on the 20 previously selected sub-networks. The selection and mutation steps are repeated until the total fitness of the new population remains constant. Finally, an optimal pruned network can be achieved.

Furthermore, because the commonly used global BN may result in sub-optimal performance [30], we updated the BN [31] to an adaptive BN to overcome this issue. For mini-batch samples of size *N*, the training data $x_{i}$, the statistics of μ, and $\sigma^{2}$ used in the adaptive BN are computed with Equations (2) and (3).

(2) $$\mu_{B}=\frac{1}{N}\sum_{i=1}^{N}x_{i}$$

(3) $$\sigma_{B}^{2}=\frac{1}{N-1}\sum_{i=1}^{N}{\left(x_{i}-\mu_{B}\right)}^{2}$$

#### 2.3.2. Fine-Tuning Based on Knowledge Distillation with MSE Loss

**Figure 4:**
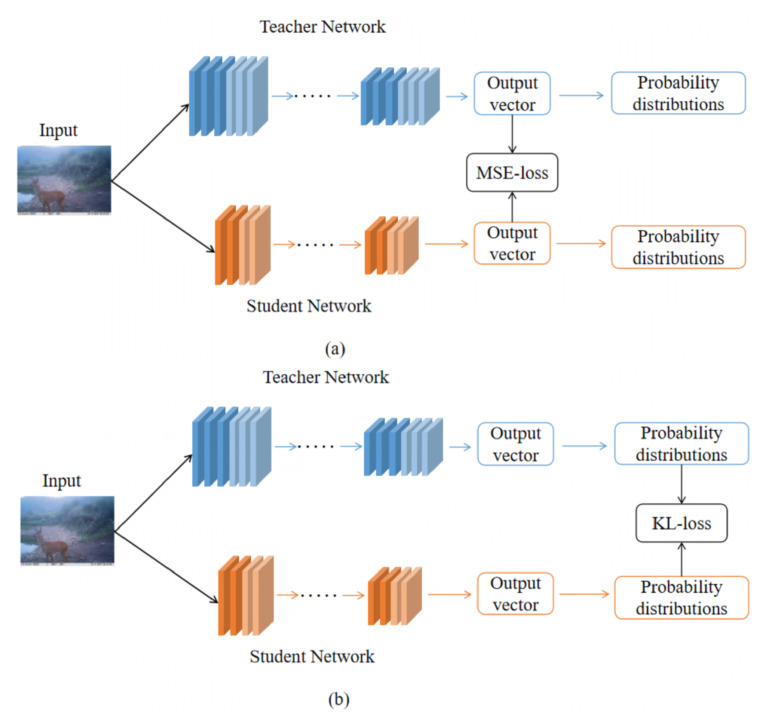
Fine-tuning based on knowledge distillation. (**a**) indicates mean square error (MSE) distillation loss and (**b**) indicates hard soft kullback-leibler (KL) distillation loss.

Knowledge distillation is introduced to improve the accuracy of the student model. As shown in Figure 4, we jointly train the teacher model and the student model with knowledge distillation loss, resulting in the student model’s distribution being comparable to that of the teacher model.

The knowledge distillation with MSE loss is introduced to fine-tune the pruned model (see Figure 4a). The MSE loss [32] is applied to compute the difference between the output distributions of the teacher and student models. For comparison, two types of KL divergence-based knowledge distillation (cf. Figure 4b) are included: knowledge distillation with soft KL (KD-SKL) and knowledge distillation with hard KL (KD-HKL). In KL divergence-based knowledge distillation, the teacher model imposes a supervision signal on the student model. KD-SKL adopts probability scores as a supervision signal, whereas KD-HKL uses one-hot pseudo labels, resulting in two different KL divergence losses.

Following knowledge distillation, the fine-tuned student model may be viewed as a lightweight wildlife recognition model with relatively high performance.

## 3. Experiments and Results

### 3.1. Experiments Setup

Because ResNet-50 provides excellent performance in wildlife image recognition [6], we built the ResNet50-based wildlife recognition model as the teacher model. During training, the dataset was divided into training and test sets in a 7:3 ratio. All images were downsized to 448×448 before being fed to the model.

Table 2 shows the software and hardware used in all experiments. All of the models’ main training settings are the same, as indicated in Table 3.

### 3.2. Evaluation Metrics

The following metrics are introduced to evaluate the performance of the models:

(1) Classification performance

The accuracy calculated by Equation (4) is used to evaluate the classification performance.

(4) $$ACC_{cls}=\frac{\sum_{i=1}^{c}TP_{i}}{\sum_{i=1}^{c}\left(TP_{i}+FP_{i}\right)}$$

where $TP_{i}$ (true positive) represents the number of correctly classified images of the *i*th class and $FP_{i}$ (false positive) represents the number of misclassified images of the *i*th class. *C* is the total number of categories.

(2) Performance of mitigating shortcut learning

**Figure 5:**
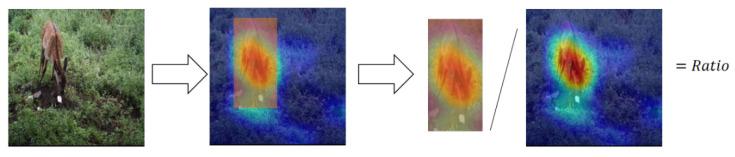
Schematic diagram of FRoH.

A heatmap can indicate where a model focuses on during classification [33]. We propose the foreground ratio of the heatmap (FRoH) to evaluate the performance of mitigating shortcut learning. The FRoH is defined by Equation (5):

(5) $$FRoH=\frac{H_{\mathrm{box}\;}}{H_{\mathrm{total}\;}}$$

where $H_{\mathrm{total}\;}$ is the sum of the thermal values of all pixels in the heatmap, and $H_{\mathrm{box}\;}$ is the sum of the thermal values of the pixels in the labeled bounding box. Figure 5 shows an intuitive schematic diagram of FRoH. FRoH indicates how much the model focuses on the targets (wildlife). The higher the FRoH value, the more the model is focused on the wildlife, implying that the model classifies images primarily on wildlife features rather than background and that shortcut learning is mitigated.

(3) Performance of model compression

The number of parameters, number of calculations, and FPS (frames per second) are introduced to quantify the performance of model compression. Equations (6) and (7) specify the number of parameters and number of calculations of the convolutional layer, respectively.

(6) $$\;\mathrm{params}\;=c_{\mathrm{out}\;}\times \left(K^{2}\times c_{in}\right)$$

(7) $$\mathrm{FLOPs}=2HW\left(c_{in}K^{2}+1\right)c_{out}$$

where $C_{\mathrm{out}\;}$ and $C_{\mathrm{in}\;}$ represent the number of output and input channels, respectively. *H* and *W* represent the height and width of the input feature map, respectively. *K* is the size of the convolution kernel.

### 3.3. Experiments Results of Mitigating Shortcut Learning

#### 3.3.1. Comparison Experiments with Different Data Augmentation Methods

Using the ResNet50-based wildlife recognition model as a baseline, five different data augmentation methods were compared, including repeat sampling, cutout [34], IS, RBS and the mixed method. The results shown in Table 4 indicate that our proposed method achieves the best performance, with $ACC_{cls}$ and FRoH values of 91.23% and 44.71%, respectively. Surprisingly, the results using repeat sampling are somewhat lower than the baseline. This is because simple random repeat sampling has no effect on the overall distribution of the dataset. The strong coupling between wildlife and background remains, resulting in no improvement in recognition accuracy. The cutout method has a higher FRoH score than the baseline, implying that the cutout method can decrease the coupling between wildlife and background to some extent. However, the cutout method has a slightly lower $ACC_{cls}$ value than that of the baseline. Because the cutout method’s optional suppression regions cover the whole image, including the wildlife region, the wildlife feature may be lost. Both IS and RBS perform better than the baseline. IS outperforms RBS because it actively diversifies the background, resulting in more abundant variety than simply suppressing a portion of the background. As expected, the mixed data augmentation method outperforms IS and RBS.

#### 3.3.2. Visualization Analysis

**Figure 6:**
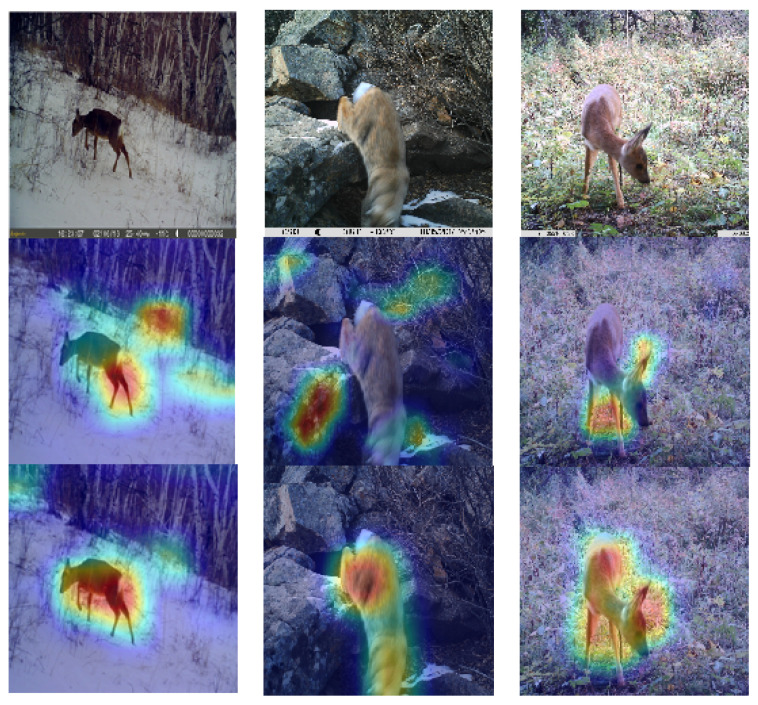
Heatmaps of typical images. The first row contains the input images, the second row contains the heatmaps of images without the mixed augmentation method, and the third row contains the heatmaps of images with mixed augmentation method.

To conduct a more in-depth analysis of the performance improvement brought by the mixed data augmentation method, we calculated a heatmap using gradient-weighted class activation mapping (Grad-CAM) [35]. Heatmaps of example images with or without mixed data augmentation method are illustrated in Figure 6. It can be observed that with mixed data augmentation, the trained wildlife recognition model can precisely focus on the wildlife (cf. last row in Figure 6). However, in the absence of mixed data augmentation, the trained wildlife recognition model is easily disturbed by the background or can only acquire incomplete wildlife features (see the second row in Figure 6).

**Figure 7:**
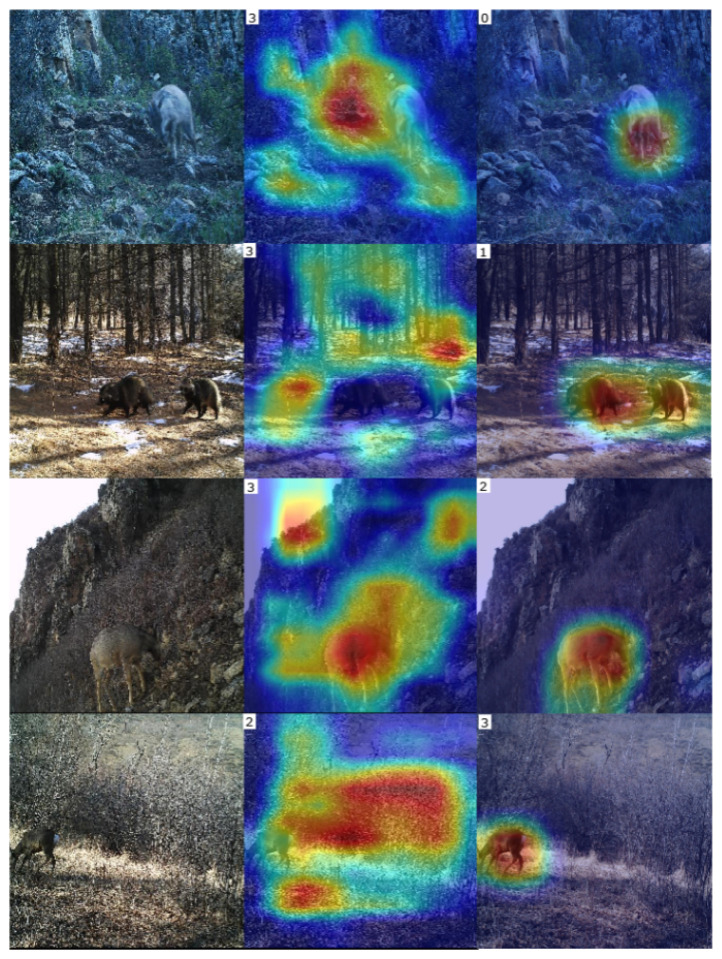
Recognition results with or without mixed augmentation method. From up to down, there are the images of goral (label 0), badger (label 1), red deer (label 2), roe deer (label 3). The first column is the input images, second column are the heatmaps of images without mixed augmentation method, the third column is the heatmaps of images with mixed augmentation method. The top left corner of each heatmap presents the predicted label.

Figure 7 shows the recognition results for example images of gorals, badgers, red deer, and roe deer. Without the mixed data augmentation, the wildlife recognition model predicted the wrong categories of wildlife (see the second column in Figure 7), which can be ascribed to an excessive focus on the background. The wildlife recognition model with the mixed data augmentation method is completely concerned with all wildlife, resulting in accurate predictions (see the third column in Figure 7).

#### 3.3.3. Class-Wise Accuracy

**Figure 8:**
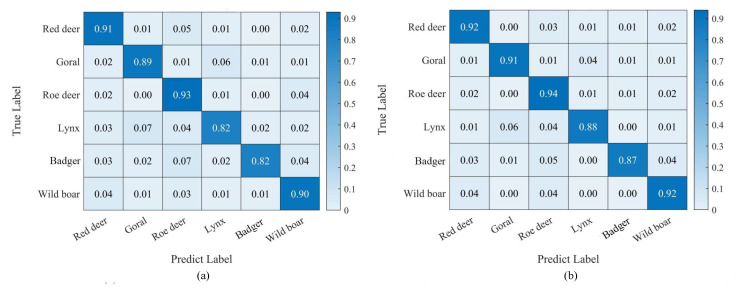
Confusion matrix. (**a**) indicates class wise accuracies of ResNet-50 and (**b**) indicates class wise accuracies of our method (+Mixed(+RBS+IS)).

Confusion matrices were computed to investigate the effect of the proposed mixed data augmentation strategy on the recognition performance of different species, as shown in Figure 8. When comparing Figure 8a,b, the mixed data augmentation strategy improved the recognition accuracy of all species. Lynxes and badgers, in particular, improved by 7.3% and 6.1%, respectively. The limited sample size and background type of these two species, together with their small size, made it easier for the model to accomplish recognition by background, and so enrichment of background variety resulted in a larger improvement.

#### 3.3.4. Performance on Other Dataset

We investigated the mixed data augmentation strategy further on the NACTI dataset. The NACTI dataset contains around 3.7 million camera trap monitoring images from five locations across the United States. We chose eleven species for comparative experiments. As with the Wildlife-6 dataset, the bounding boxes containing wildlife were annotated and used in the following experiments. Table 5 shows the results on the NACTI dataset. The $ACC_{cls}$ and FRoH values were both enhanced after utilizing the mixed data augmentation method, with the FRoH being increased to a greater extent. The improvement in $ACC_{cls}$ is rather minimal due to the excellent accuracy of the baseline and the more diverse backgrounds in the NACTI dataset.

In summary, the mixed data augmentation strategy can assist the wildlife recognition model in focusing more on the wildlife, resulting in higher classification accuracy and generalization across datasets. In other words, the shortcut learning can be mitigated.

### 3.4. Experiments Results of the Lightweight Automatic Recognition Model

#### 3.4.1. Comparison with Different Pruning Method

**Figure 9:**
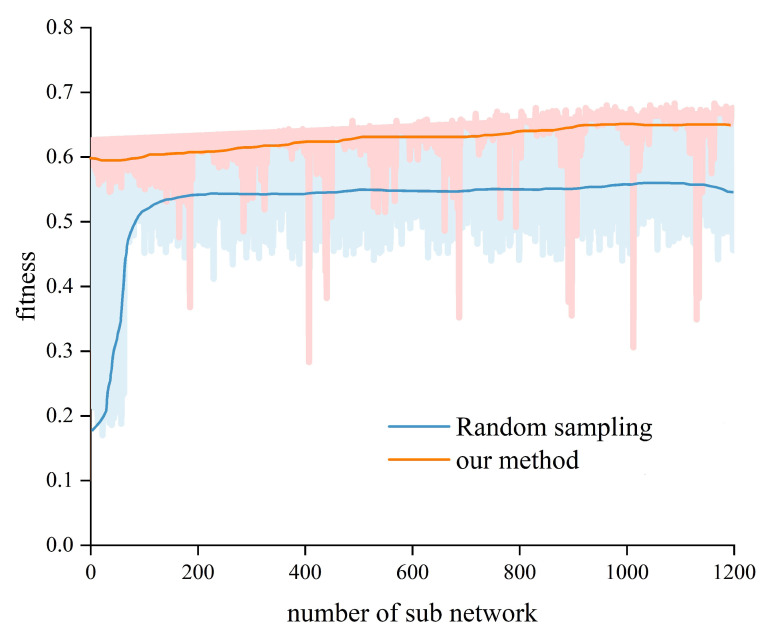
Fitness variations of the sub-networks during the search under 50 ± 5% compression ratio. The solid line shows the sliding average of the fitness (*N* = 3).

With the ResNet50-based wildlife recognition model as the teacher model, two pruning methods, the GA-ABN and random sampling algorithms, were compared. The compression ratio of FLOPs was set to 50 ± 5%. The fitness variations of the sub-networks generated by two pruning methods during the searching process are depicted in Figure 9. It can be shown that the fitness (i.e., accuracy) of the random sampling method improved significantly at first, but then almost equalized in the later search period. This is due to the lack of an optimization mechanism. Taking advantage of GA’s evolution mechanism, our method can achieve higher and higher fitness as time passes, implying a better pruning strategy. Meanwhile, the final fitness of our method is greater than that of the pruning method using random sampling.

We further fine-tuned the optimal pruned model obtained by two pruning methods. As shown in Table 6, the accuracy of the ResNet50-based model is 91.23%, the number of parameters is 23.52 M, the FLOPs is 16.48 G and the FPS is 0.85. The number of parameters of the two pruned models were reduced by almost half. Furthermore, the accuracy values of the two pruned models were decreased as expected, and the pruned model by GA-ABN achieved greater accuracy with fewer parameters than the pruned model by random sampling. Similar to the numbers of parameters, the FLOPs of both pruned models declined by almost half, resulting in higher FPS. The FPS values of both pruned models increased by nearly 62 times as compared with the ResNet50 model. Although the FLOP and FPS of our method are similar with those of random sampling, our method has a higher accuracy than random sampling.

Furthermore, the compression ratio of FLOPs was set to 25 ± 5% to verify the generalization of the proposed pruning method. As shown in Table 7, although FLOPs and FPS are the same, the accuracy of the pruned model achieved by our method is still higher than that of the pruned model using random sampling.

Altogether, the pruning method using GA-ABN can effectively reduce the number of computations in the model, speed up the model operation, and maintain relatively high accuracy under different pruning ratios, showing that the GA-ABN pruning method has excellent compression performance.

#### 3.4.2. Fine-Tuning Based on Different Knowledge Distillation Methods

Three fine-tuning strategies based on KD-MSE, KD-SKL, and KD-HKL were compared with the pruned ResNet50 model using GA-ABN as the baseline model (P-ResNet50). As shown in Table 8, all three fine-tuning strategies outperformed the P-ResNet50 model in terms of accuracy. Furthermore, the accuracy of KD-MSE was 1.88%, 0.66%, and 1.39% higher than those of P-ResNet50, KD-SKL, and KD-HKL, respectively, indicating that KD-MSE is the most effective fine-tuning method. The advantage of KD-MSE originates from the fact that it enables the student network to inherit the output distribution of the teacher network via an explicit MSE constraint. KD-HKL, in particular, performs worse than KD-SKL, which we attribute to possible noise introduced by a hard supervision signal during training.

## 4. Discussion

Wildlife camera trap image recognition based on deep learning offers enormous potential, but it is also challenging due to the complexity of the wild environment [3]. Our work investigates a mixed data augmentation technique for mitigating shortcut learning of deep learning models caused by similar backgrounds in certain images from the same camera trap. Intelligent recognition implemented directly on edge devices can improve the effectiveness of wildlife monitoring [36], we further propose a novel model compression strategy that integrates pruning and knowledge distillation to develop lightweight wildlife recognition models applicable to camera traps.

### 4.1. Overcoming Shortcut Learning with Data Augmentation

Overcoming shortcut learning can enhance model generalization performance. However, to the best of the authors’ knowledge, no work has focused on shortcut learning in camera trap image recognition. Because dataset distribution has a direct influence on deep neural network learning, data distribution variation has the potential to alleviate shortcut learning [10]. Several data augmentation strategies, which can vary the data distribution, have been employed to alleviate shortcut learning [11].

Diversifying the background in camera trap images can help distinguish the foreground (wildlife) and background and prevent the recognition model from shortcut learning. Given that repeat sampling has no effect on data distribution, it is therefore not surprising that the recognition performance has not been improved. The cutout method modifies the data distribution using random masks and improves the FRoH score compared to the baseline, but it may also suppress the foreground (wildlife) feature, which lowers the $ACC_{cls}$ in comparison to the baseline. IS and RBS both retain foreground features while modifying the data distribution, achieve higher FRoHs than the baseline, guide the model to focus more on the foreground, and avoid shortcut learning very well. As expected, a mixed data augmentation approach that combines IS and RBS performs the best. Using the Wildlife-6 dataset, our recognition accuracy (91.23%) is higher than that of the literature [6] (90.2%), demonstrating that our technique is effective.

### 4.2. Lightweight Wildlife Recognition Model

Because camera traps have limited computational capacity and memory, lightweight models are required for deep learning-based automatic wildlife recognition in camera traps [36]. Model compression strategies based on model pruning and knowledge distillation are the dominant approaches for developing lightweight models [37,38]. In knowledge distillation methods, selecting a suitable student model is crucial. Model pruning can produce beneficial tiny networks, but it can also reduce accuracy [20,21]. A potential strategy appears to be model pruning to generate student models and knowledge distillation for fine-tuning [25].

The key to structured pruning is to search for redundant channels or layers, which is an optimization problem. GA can effectively avoid falling into local optima and has been utilized in model pruning [38]. Experiments show that the proposed GA-ABN can search for and achieve sub-networks with higher final fitness scores more quickly when compared to random sampling algorithms. The GA-ABN method can achieve a higher accuracy than random sampling algorithms. The greater the compression rate, the more obvious the benefit. With a compression rate of 50 ± 5%, the FPS of our method reaches 53.49, about 63 times that of ResNet50, which is sufficient for provision of real-time results (30 FPS or more) [39].

Furthermore, we introduced three kinds of knowledge distillation methods, KD-MSE, KD-SKL and KD-HKL to fine-tune the pruned model to improve accuracy. MSE loss can make the student model learn the output distribution of the teacher model more intuitively [40], which makes the KD-MSE achieved the highest accuracy improvement among three methods.

Finally, a lightweight wildlife recognition model with high accuracy but a massively reduced number of parameters and a significantly lower calculation can be achieved. The proposed lightweight wildlife recognition model design method has the potential to enable the application of edge intelligence. Future research will investigate the lightweight model’s performance on embedded devices.

## 5. Conclusions

This study proposed a lightweight wildlife recognition model design method that combines a mixed data augmentation strategy, as well as a model compression method with integrated pruning and knowledge distillation.

The mixed data augmentation method, combined with IS and RBS, was introduced to modify the data distribution of the dataset, guiding the model to focus more on the wildlife feature. The ACCcls and FRoH were both improved after using the mixed data augmentation method, demonstrating that shortcut learning was mitigated.

Furthermore, the lightweight model was constructed using an integrated method that first employed the structural pruning technique based on GA-ABN, then fine-tuned the pruned model with KD-MSE. Using GA-ABN pruning and a compression rate of 50 ± 5%, the number of model parameters reduced by 57.4%, FLOPs decreased by 46.1%, FPS improved to 62.9 times, and accuracy decreased by just 4.73%. After fine-tuning with KD-MSE, the accuracy of the model improved by 2.12%.

Overall, this study provides a novel method for developing lightweight wildlife recognition models, which can result in lightweight models with relatively high accuracy and significantly decreased computing cost. Our method has important practical implications for utilizing deep learning models for wildlife monitoring on edge intelligent devices. This can help to support long-term wildlife monitoring, biodiversity resource assessments, and ecological conservation.

In the future, we intend to deploy our lightweight model to camera traps and augment them with wireless communication capabilities to create a real-time wildlife monitoring system. The performance of proposed system will be evaluated in the wild.

## Figures and Tables

**Table 1:** Details of Wildlife-6.

Species	Number of Images
Red deer	1094
Goral	761
Roe deer	1310
Lynx	377
Badger	190
Wild boar	906
Total	4638

**Table 2:** Setup of the experiments.

Test Environment	Type
System	Ubuntu16.04
Graphics card	Nvidia 1080 ti (11 GB/Nvidia)
CPU	Intel Core i5-9400F @ 2.9GHz
RAM	32 GB
Framework	Pytorch1.4.1
Programming language	Python3.6

**Table 3:** Training settings.

Item	Value or Method
Optimizer	SGD
Momentum	0.9
Initial learning rate	0.0001
Learning rate decay	cosine decay
Batch size	64
Iterative scheme	early stop, patience = 50

SGD = stochastic gradient descent.

**Table 4:** Results of different data augmentation methods.

Methods	${\mathrm{Acc}}_{\mathrm{cls}\;}$	FRoH
ResNet-50	89.76%	43.40%
+ Repeat sampling	88.23%	43.36%
+ Cutout	89.03%	43.83%
+RBS	90.13%	43.57%
+ IS	90.72%	43.95%
+Mixed (+RBS + IS)	91.23%	44.71%

RBS = regional background suppression; IS = image synthesis.

**Table 5:** Results of the NACTI dataset.

Methods	${\mathrm{Acc}}_{\mathrm{cls}\;}$	FRoH
ResNet-50	99.21%	46.48%
+Mixed (+RBS + IS)	99.24%	49.35%

RBS = regional background suppression; IS = image synthesis.

**Table 6:** Performances of ResNet50 and the pruned models with compression ratio of 50 ± 5%.

	ResNet50	Random Sampling	Our Method
Accuracy	91.23%	84.90%	86.50%
Parameters	23.52 M	10.65 M	10.01 M
FLOPs	16.48 G	8.85 G	8.89 G
FPS	0.85	53.2	53.49

FLOPs = floating-point operations per second; FPS = frames per second.

**Table 7:** Performances of the pruned models with compression ratio of 25 ± 5%.

	Random Sampling	Our Method
Accuracy	83.80%	86.20%
Parameters	5.65 M	5.65 M
FLOPs	4.73 G	4.73 G
FPS	56.62	56.62

FLOPs = floating-point operations per second; FPS = frames per second.

**Table 8:** Result of different fine-tuning framework on our dataset.

Method	Accuracy
P-ResNet50	86.50%
KD-SKL	87.72%
KD-HKL	86.99%
KD-MSE	88.38%

KD-SKL = knowledge distillation with soft kullback-leibler; KD-HKL = knowledge distillation with hard kullbackleibler; KD-MSE = knowledge distillation with mean square error.

## Data Availability

The datasets used in this study are available from the corresponding author on reasonable request.
